# Supplementary material for: A Method for Estimating the Entropy of Time Series Using Artificial Neural Networks
Source: Entropy (Basel). 2021 Oct 29;23(11):1432. doi: 10.3390/e23111432 (PMC8621949; doi:10.3390/e23111432)
Supplement: Supplementary file 1 [file entropy-23-01432-s001.zip › Manual.pdf]

Manual

The “NNetEn calculator 1.0.0.0” software calculates the entropy of time series using the method presented in

Velichko, A.; Heidari, H. A Method for Estimating the Entropy of Time Series Using Artificial Neural Networks. *Entropy* **2021**.

The program operates in the OS Windows environment and consists of the following files and folders:

• “NNetEn.exe” - executable file of the software;

• “MNIST_10” - the folder contains the MNIST database downloaded from Jan Lekun's site;

http://yann.lecun.com/exdb/mnist/index.html;

• “T_pattern” - the folder contains the T-Pattern;

• “Results” - the folder contains a list of calculation results.

1. File “NNetEn.exe” launches the program.

2. To load a file with data, click on the “Open file” button. Data should be presented in columns separated by tabs. Examples of data files:

Data_example 1 (Logistic map r1 r2 r3 r4) .txt,

Data_example 2 (Random discrete map) .txt,

Data_example 3 (Binary discrete map) .txt.

After loading, the first 10 lines of the file are visible in the corresponding field of the program interface.

3. Select the column of the time series in the “Column” field, press the “Show” button. The series is displayed in the “Time series xn” field.

4. Select the length of the series in the field “N =”.

5. Set the number of epochs in the “Step 6:” field.

6. Start the calculation with the “START” button. The results are displayed in the “Result” field.

7. To process all series, press the “START of All columns” button.

8. The calculation can be stopped by pressing the “STOP” button.

The program is single-threaded. Several programs can be run at the same time with different input data for more efficient use of processor power.

When using this software, the reference to the original contribution of the authors is compulsory.

Please send proposals for program improvements and research collaboration to velichko@petrsu.ru indicating “NNetEn calculator” in the subject field.

Velichko A.
